# Evaluating the Effectiveness of MRI Versus CT in Identifying Retroperitoneal Lymph Node Metastasis in Testicular Cancer: A Systematic Review and Meta-Analysis

**DOI:** 10.7759/cureus.76911

**Published:** 2025-01-04

**Authors:** Reem M Aldossari, Muaiyad Alkindi, Othman M Alshamrani, Raghad E Abdullah, Shaden M Alosaimi, Taghreed A Alsaleh, Joud M Babegi, Mohammed A Altabsh, Majed J Alhazmi, Salman M Alfawaz, Budur A Qasem, Hana Alfaleh

**Affiliations:** 1 College of Medicine, King Saud University, Riyadh, SAU; 2 Division of Diagnostic Radiology, Oman Medical Specialty Board, Muscat, OMN; 3 College of Medicine, King Saud Bin Abdulaziz University for Health Sciences, Riyadh, SAU; 4 Division of Diagnostic Radiology, King Fahad General Hospital, Jeddah, SAU; 5 College of Medicine, Taif University, Taif, SAU; 6 Division of Diagnostic Radiology, King Khalid Hospital, Najran, SAU; 7 Diagnostic Radiology Technology Department, College of Nursing and Health Sciences, Jazan University, Jazan, SAU; 8 Department of Urology, King Abdulaziz University, Jeddah, SAU; 9 College of Medicine, Mansoura University, Mansoura, EGY; 10 Physics Department, College of Applied Sciences, Umm Al-Qura University, Makkah, SAU; 11 Department of Medical Imaging, King Abdulaziz Medical City, Riyadh, SAU

**Keywords:** computed tomography, diagnostic accuracy, magnetic resonance imaging, retroperitoneal lymph nodes, testicular cancer

## Abstract

Testicular cancer, while rare, is a common malignancy among males aged 15-45 years and often spreads to retroperitoneal lymph nodes. This systematic review and meta-analysis compares the diagnostic accuracy of magnetic resonance imaging (MRI) and computed tomography (CT) in detecting retroperitoneal lymph node metastasis. A comprehensive search was conducted up to January 25, 2024, using PubMed, MEDLINE, Web of Science, and Google Scholar. Studies comparing MRI and CT for detecting retroperitoneal lymph node involvement in adult males with testicular neoplasms were included. Data extraction covered study design, sample size, demographics, imaging techniques, and diagnostic outcomes. The Newcastle-Ottawa Scale was used to assess the risk of bias, and a random-effects model was applied for the meta-analysis. A total of 618 articles were identified, with four meeting the inclusion criteria. The studies reported high sensitivity for both MRI and CT, with MRI sensitivities ranging from 97% to 100% and CT sensitivities from 96% to 100%. Specificity findings were variable, with some studies suggesting similar or slightly higher values for MRI. Meta-analysis of three studies revealed no significant difference between MRI and CT in detecting retroperitoneal lymph node metastasis, with an odds ratio of 1.00 (95% CI: 0.54 to 1.86) and minimal heterogeneity (I² = 0%). These findings suggest both MRI and CT demonstrate comparable diagnostic accuracy in detecting retroperitoneal lymph node metastasis in testicular cancer. While MRI avoids ionizing radiation, it requires expert interpretation and is more costly, limiting its accessibility in some settings.

## Introduction and background

Testicular cancer is a rare malignancy, accounting for only 1% of all male cancers. However, it is a relatively common malignancy among males aged 15-45 years [1,2]. Globally, the incidence of testicular cancer has been on the rise, particularly within the young age group [1-7]. This increase in incidence is contrasted by a significant decline in mortality rates, which can be attributed to advances in early diagnostic techniques and the efficacy of current treatment modalities [1,3,4].

Testicular cancer most commonly metastasizes via lymphatic drainage, with retroperitoneal lymph nodes being the most common site for metastatic spread [8-11]. Accurate staging and monitoring of retroperitoneal lymph node involvement are critical for the effective management and treatment of testicular cancer. Contrast-enhanced computed tomography (CT) of the abdomen and pelvis has traditionally been considered the standard imaging modality for identifying retroperitoneal lymph node metastasis due to its detailed anatomical resolution and widespread availability [8,12].

However, the use of CT imaging exposes patients to ionizing radiation, which is a considerable concern given the relatively young age of testicular cancer patients and the extensive follow-up imaging required [13-16]. As a result, magnetic resonance imaging (MRI) has been proposed as an alternative imaging modality for the evaluation of retroperitoneal lymphadenopathy, offering high-resolution images without the associated radiation risks [8,17]. Recent guidelines by the European Association of Urology (EAU), National Comprehensive Cancer Network (NCCN), and European Society for Medical Oncology (ESMO) have recommended the use of MRI instead of CT for the follow-up of testicular cancer patients under certain clinical conditions [18-20].

Therefore, the objective of this systematic review is to analyze the available evidence on the diagnostic accuracy of MRI versus CT in identifying retroperitoneal lymph node metastases in adult males with testicular cancer. By focusing on outcomes such as sensitivity, specificity, and overall accuracy, this review seeks to provide a comprehensive assessment of these imaging modalities to promote clinical practices, enhance patient safety, and establish optimized imaging standards for the follow-up and management of testicular cancer.

## Review

Materials and methods

Literature Search Strategy

This systematic review was prospectively registered in the International Prospective Register of Systematic Reviews (PROSPERO) (ID: CRD42024509557) and conducted in adherence to the Preferred Reporting Items for Systematic Reviews and Meta-Analyses (PRISMA) guidelines [21]. The lead researcher meticulously designed the systematic review protocol, which was subsequently approved by the entire research team.

Without any restrictions, a comprehensive electronic search was performed up to January 25, 2024, across multiple databases, including PubMed, MEDLINE, Web of Science, and Google Scholar. The following search terms were used to identify relevant studies: (testicular cancer OR testicular neoplasms OR testicular tumor OR testicular malignancy OR testicular carcinoma OR Seminoma) AND (MRI OR magnetic resonance imaging OR MR imaging OR magnetic resonance) AND (CT OR computed tomography OR CT scan OR computerized tomography OR X-Ray Computed) AND (lymph node OR lymph node metastasis OR lymph node involvement OR spread to lymph nodes OR lymphatic metastasis).

Inclusion and Exclusion Criteria

The review included studies published in English that compared the accuracy of MRI and CT in identifying retroperitoneal lymph node metastasis of testicular germ cell tumors in human males aged over 18 years. The study types considered for inclusion were randomized clinical trials, prospective and retrospective cohort studies, case-control studies, and cross-sectional studies. There were no restrictions on the publication date, and all studies published up to the search date were included. Studies were excluded if they did not compare the accuracy of MRI versus CT in identifying retroperitoneal lymph node metastasis of testicular germ cell tumors, lacked sufficient details regarding the assessed outcomes, or did not fit the specified design types (e.g., case reports, case series, editorials, letters, commentaries, reviews). In addition, studies involving participants younger than 18 years, non-human studies, and non-English studies were excluded.

Selection of Articles and Data Extraction

All studies identified from the primary search were imported into Mendeley Reference Manager (version 2.120.3) for deduplication and subsequently uploaded to Rayyan, a web-based platform used collaboratively by reviewers to screen titles and abstracts based on inclusion and exclusion criteria [22]. Four reviewers independently screened the titles and abstracts to identify studies meeting the inclusion criteria. Full texts of studies that passed the title and abstract screening were further assessed for eligibility. Data extraction from the final included studies was conducted by another group of four reviewers, encompassing the following components: title, first author, year of publication, country of origin, study design type, sample size, patient mean age, testicular cancer type and stage, imaging technique used, reference standard test, MRI and CT machine type, evaluator experience, MRI and CT sensitivity, specificity, positive predictive value (PPV), negative predictive value (NPV) for identifying retroperitoneal lymph node metastasis, and lymph node size cut-off value. Any disagreements or inconsistencies during the study selection process or data extraction were resolved by a fifth reviewer.

Bias and Quality Assessment

The quality of the included studies and the risk of bias were assessed using the Newcastle-Ottawa Scale (NOS) [23]. The NOS evaluates methodological quality through eight items distributed across three categories: selection, comparability, and outcomes. The scale ranges from 0 to 9, with scores interpreted as follows: a score of 7-9 indicates a high-quality study with a low risk of bias, a score of 4-6 indicates a fair-quality study with a moderate risk of bias, and a score of 0-3 indicates a low-quality study with a high risk of bias. Two reviewers independently assessed the risk of bias for eligible case-control and cohort studies. A third reviewer reviewed and verified the assessments to ensure consistency and accuracy.

Meta-Analysis

The meta-analysis was conducted using a random-effects model to account for both within-study and between-study variability. The odds ratio and corresponding 95% confidence interval (CI) were calculated to compare the diagnostic accuracy of MRI and CT. A forest plot was generated to visualize these results. Given the absence of heterogeneity among the studies (I² = 0%), further subgroup analysis was deemed unnecessary. All analyses were performed using Review Manager software (version 8.5.1).

At the manuscript preparation stage, ChatGPT (version 4.0, OpenAI, San Francisco, California) was utilized to assist in rephrasing the text throughout the manuscript, ensuring clarity and coherence. The authors were fully responsible for the content, ensuring that the manuscript represents their original ideas and conclusions.

Results

Literature Findings

**Figure 1:**
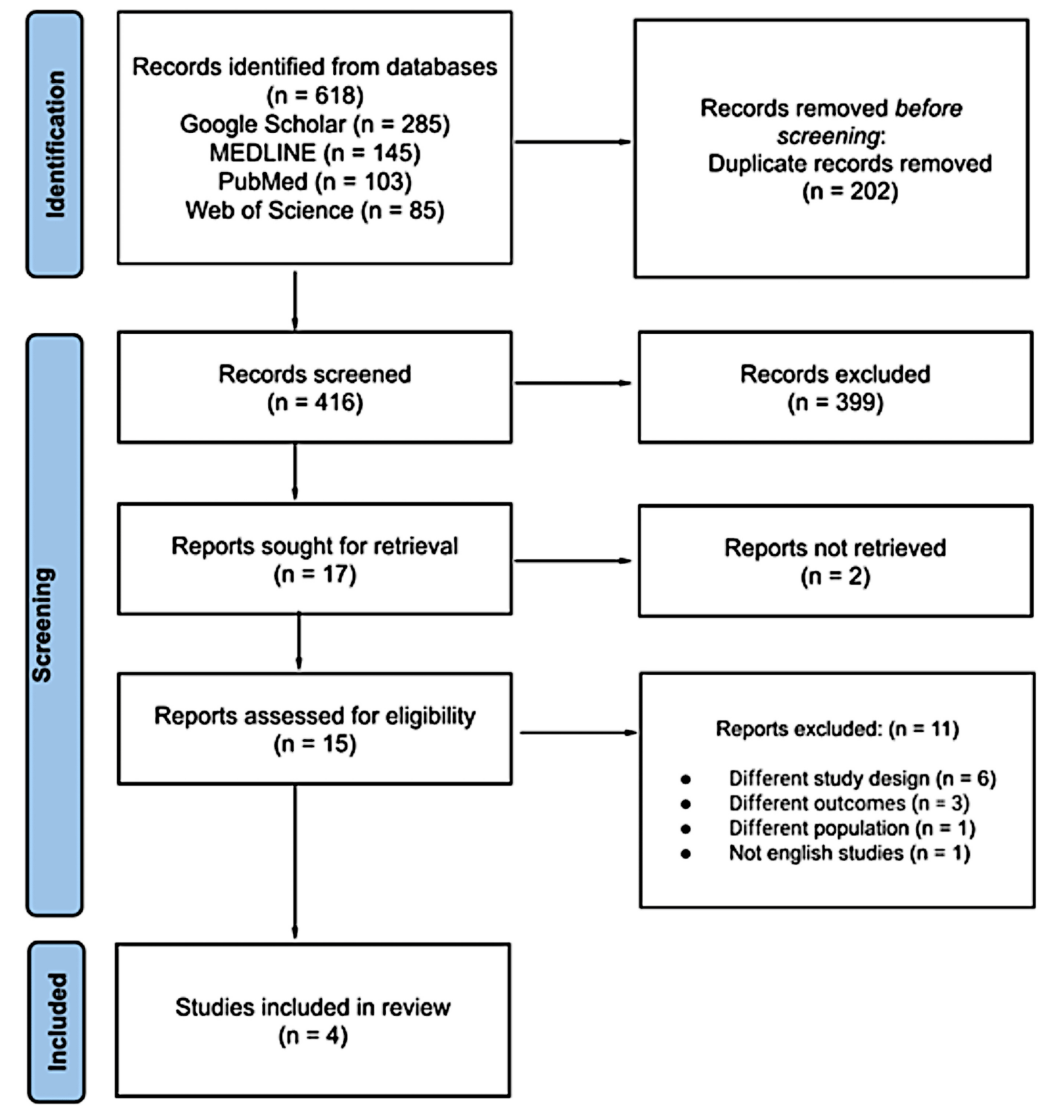
PRISMA flow chart for study selection. This PRISMA (Preferred Reporting Items for Systematic Reviews and Meta-Analyses) flow chart details the study selection process, including records identified from databases, screened, and the final studies included in the systematic review.

A total of 618 articles were identified through the search strategy: 145 from MEDLINE, 103 from PubMed, 85 from Web of Science, and 285 from Google Scholar. After removing duplicates, 416 articles remained for screening. We retrieved 15 full-text publications for further assessment. Following the application of predefined inclusion and exclusion criteria, four articles were eligible and included in the systematic review (Figure 1). The exclusion of 11 articles was due to the following reasons: different study design (n = 6), different outcome (n = 3), different population (n = 1), and non-English study (n = 1).

Characteristics of the Included Studies

Four studies comparing MRI and CT in identifying retroperitoneal lymph node metastasis in testicular cancer were selected [24-27]. Three of these studies were prospective cohort studies [24-26], while one was a case-control study [27]. The studies were published in Belgium [26], Finland [27], the UK [24], and Denmark [25]. Across the studies, a total of 229 patients with testicular cancer were included, of whom 179 had retroperitoneal lymph node metastasis. Testicular cancer stages, ranging from I to IV, were reported in two studies [26,27]. The age distribution of participants varied from 18 to 65 years. All studies used CT as a reference standard test [24,25,27], except for one study [26], which used histopathologic examination for three patients and the best valuable comparator (BVC) for the remaining 40 patients. Criteria for abnormal lymph nodes were specified in three studies [24,26,27], typically including a size larger than 7-10 mm, loss of normal shape, and irregular outlines. A detailed breakdown of each study is provided in Table 1.

**Table 1:** Characteristics of the included studies. This table presents comprehensive details of the included studies, such as country, study type, diagnosis, number of patients, mean age, imaging techniques (magnetic resonance imaging (MRI) and computed tomography (CT)), and other relevant data. Abbreviations: CT: computed tomography, MRI: magnetic resonance imaging, DWI: diffusion-weighted imaging, DWIBS: diffusion-weighted imaging with background suppression, BVC: best valuable comparator, pts: patients, N/A: not available.

Study	Country	Study type	Diagnosis	Number of patients	Mean age	Imaging technique used	Reference standard test	Evaluator expertise	MRI machine	CT machine	Lymph node size cut-off value
Sohaib et al., 2009 [24]	United Kingdom	Prospective study	Testicular germ cell tumor	52	34	MRI and contrast-enhanced CT	CT	Both CT and MRI by two experienced radiologists (10 years of experience) and one radiologist (1 year of experience)	A Philips Intera 1.5T MRI	A GE LightSpeed 16 Slice CT	10 mm in short axis
Laukka et al., 2020 [27]	Finland	Prospective case-control	Testicular germ cell tumor Stage I, II, III, IV	50	33	MRI with DWI and contrast-enhanced CT	CT	Both CT and MRI by one experienced radiologist	A 1.5T GE MRI	5 different spiral CT machines provided by Siemens, Toshiba, and GE Medical systems	7 mm in short axis
Larsen et al., 2022 [25]	Denmark	Prospective non-inferiority	Testicular germ cell tumor	84	N/A	MRI with DWIBS and contrast-enhanced CT	CT	CT by two oncological radiologists (18/30 years of experience) and MRI by two oncological radiologists (14/23 years of experience)	A Philips Ingenia 1.5T	N/A	N/A
Pasoglou et al., 2022 [26]	Belgium	Prospective study	Testicular germ cell cancer stage I, II, III	43	35	Whole-body MRI and contrast-enhanced thoraco-abdominopelvic CT	Histopathologic examination (3 pts) and BVC (40 pts)	Both CT and MRI by two experienced radiologists (15/13 years of experience)	A Philips Ingenia (32 pts) and A General Electric Signa Premier (11 pts)	An IQON® Spectral CT scanner	10 mm in short axis

Sensitivity and Specificity

Larsen et al. [25] and Laukka et al. [27] reported a 98% sensitivity for MRI, while Laukka et al. found the sensitivity for CT to be 96%. Sohaib et al. [24] observed 97% sensitivity for MRI when interpreted by an experienced radiologist, compared to 80% with a non-experienced radiologist. Pasoglou et al. [26] observed a 100% sensitivity for both MRI and CT. Regarding specificity, Laukka et al. [27] and Pasoglou et al. [26] reported 100% specificity for MRI, with CT specificity at 75% in Laukka et al. [27] and 100% in Pasoglou et al. [26]. However, Larsen et al. [25] found MRI specificity to be 75% compared to CT. Sohaib et al. [24] focused on lesion-level analysis without detailed patient-level results. A detailed breakdown of the results is in Table 2.

**Table 2:** Diagnostic outcomes of the included studies. This table outlines the sensitivity, specificity, positive predictive value (PPV), and negative predictive value (NPV) for MRI and CT in identifying retroperitoneal lymph node metastasis in testicular cancer, demonstrating the efficacy of each imaging technique. Abbreviations: CI: confidence interval, CT: computed tomography, MRI: magnetic resonance imaging, PPV: positive predictive value, NPV: negative predictive value, ER: experienced radiologist, NER: non-experienced radiologist, N/A: not available.

Study	Sensitivity (95% CI)		Specificity (95% CI)		PPV (95% CI)		NPV (95% CI)	
	MRI	CT	MRI	CT	MRI	CT	MRI	CT
On the patient level								
Sohaib et al., 2009 [24]	ER: 97% (80-100%) NER: 80% (61-92%)	N/A	N/A	N/A	N/A	N/A	N/A	N/A
Laukka et al., 2020 [27]	98% (89-100%)	96%	100%	75%	100%	98%	80%	60%
Larsen et al., 2022 [25]	98% (91-100%)	N/A	75% (19-99%)	N/A	99% (93-100%)	N/A	60% (15-95%)	N/A
Pasoglou et al., 2022 [26]	100% (85-100%)	100% (85-100%)	100% (84-100%)	100% (84-100%)	100%	100%	100%	100%
On the lesion level								
Sohaib et al., 2009 [24]	ER: 96% (87-99%) NER: 78% (65-88%)	ER: 96% (87-99%); NER: 82% (70-90%)	ER: 100% NER: 91%	ER: 100%; NER: 96%	ER: 100%; NER: 95%	ER: 100%; NER: 98%	ER: 92%; NER: 66%	ER: 92%; NER: 71%
Larsen et al., 2022 [25]	99% (97-100%)	98% (96-100%)	78% (69-86%)	88% (78-95%)	93% (89-96%)	97% (94-99%)	95% (88-99%)	92% (83-98%)

Bias and Quality Assessment Results

The risk of bias was assessed using the NOS [23]. All four included studies were classified as high-quality [24-27], with scores ranging from 8 to 9, indicating a low risk of bias. A detailed breakdown of the assessment results is presented in Table 3.

**Table 3:** Quality assessment of the included studies using the Newcastle-Ottawa scale. This table assesses the quality of included studies using the Newcastle-Ottawa Scale (NOS), evaluating selection, comparability, and outcome measures, along with the risk of bias and overall quality score. Abbreviations: Cohort Studies: Q1–Q4: Representativeness of the exposed cohort, selection of the non-exposed cohort, ascertainment of exposure, outcome not present at the start; Q5: Comparability of cohorts; Q6–Q8: Assessment of outcome, duration of follow-up, adequacy of follow-up. Case-Control Studies: Q1–Q4: Adequacy of case definition, representativeness of cases, selection of controls, definition of controls; Q5: Comparability of cases and controls; Q6–Q8: Ascertainment of exposure, same method of ascertainment, non-response rate.

Study	Selection				Comparability	Outcome			Quality score	Risk of bias (0-3: high, 4-6: moderate, 7-9: low)
	Q1	Q2	Q3	Q4	Q5	Q6	Q7	Q8		
Cohort studies										
Sohaib et al., 2009 [24]	*	*	*	No	**	*	*	*	High-quality study (8)	Low risk
Larsen et al., 2022 [25]	*	*	*	No	**	*	*	*	High-quality study (8)	Low risk
Pasoglou et al., 2022 [26]	*	*	*	No	**	*	*	*	High-quality study (8)	Low risk
Case-control studies										
Laukka et al., 2020 [27]	*	*	*	*	**	*	*	*	High-quality study (9)	Low risk

Meta-Analysis Results

**Figure 2:**
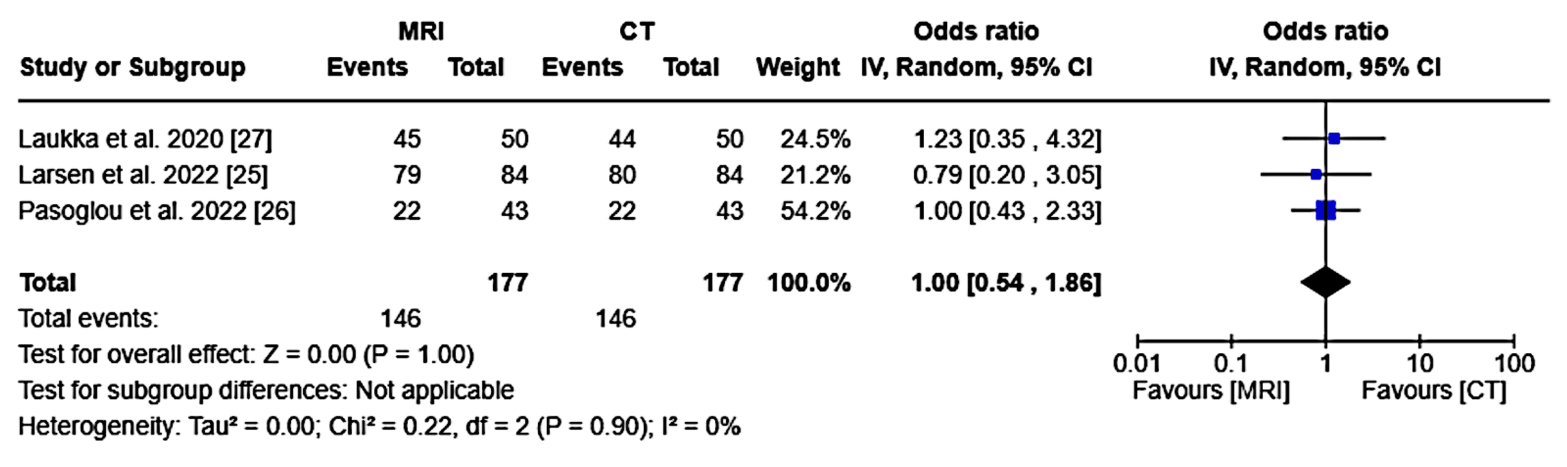
Forest plot of odds ratios for MRI vs. CT in detecting retroperitoneal lymph node metastasis. This forest plot compares the odds ratios of magnetic resonance imaging (MRI) versus computed tomography (CT) in detecting retroperitoneal lymph node metastasis in testicular cancer, summarized under a random-effects model.

The meta-analysis included three studies that compared MRI and CT in identifying retroperitoneal lymph node metastasis in patients with testicular cancer [25-27], with 177 patients in each group. Both imaging modalities detected 146 positive events. The odds ratio (OR) was 1.00 (95% CI: 0.54 to 1.86), indicating no significant difference between MRI and CT (Z = 0.00, P = 1.00). Minimal heterogeneity was observed (Tau² = 0.00, Chi² = 0.22, I² = 0%), suggesting consistent findings across the studies (Figure 2).

Discussion

The relative effectiveness of MRI and CT in identifying retroperitoneal lymph node metastasis in testicular cancer has been consistently demonstrated across various studies, underscoring the robustness of these imaging modalities [24-27]. This systematic review aims to synthesize the available evidence on the diagnostic accuracy of MRI versus CT in identifying retroperitoneal lymph node metastasis in adult males with testicular cancer, focusing on outcomes such as sensitivity, specificity, and overall accuracy.

**Figure 3:**
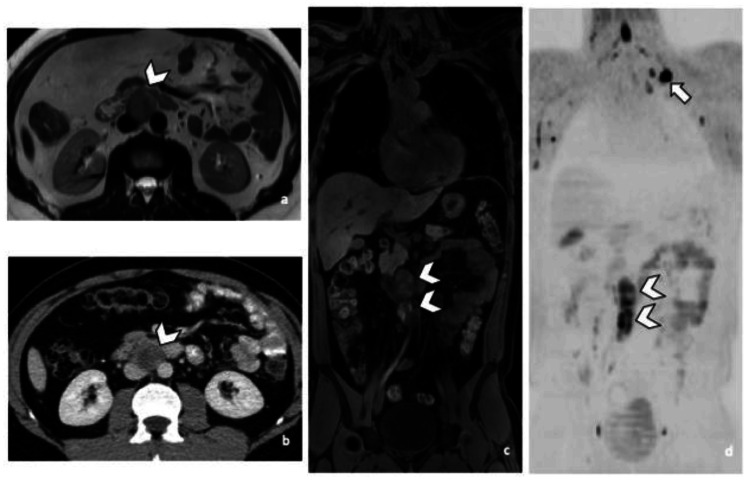
MRI and CT imaging of retroperitoneal lymph node metastases in a patient with non-seminomatous germ cell cancer. Axial T2-weighted MRI (a), axial CT with contrast (b), coronal 3D T1-weighted MRI with fat suppression (c), and diffusion-weighted MRI (d) demonstrating interaorticocaval enlarged lymph nodes (arrowheads) in a 29-year-old man. A left supraclavicular lymphadenopathy is also visible on diffusion-weighted imaging (d, arrow). Image reused from Pasoglou et al. [26] under the terms of the Creative Commons Attribution (CC BY) license.

Our review reveals that both MRI and CT demonstrate high sensitivity in detecting retroperitoneal lymph node metastasis in testicular cancer, despite minor variations reported in the literature. For instance, Sohaib et al. [24] found that MRI, when interpreted by an experienced radiologist, had a sensitivity of 97%, compared to 80% with a non-experienced radiologist. Larsen et al. [25] and Laukka et al. [27] reported an MRI sensitivity of 98%, while Laukka et al. [27] also found a CT sensitivity of 96%. In addition, Pasoglou et al. [26] reported exceptionally high sensitivity, with both MRI and CT achieving 100% in the diagnosis of metastatic disease (Figure 3).

Regarding specificity, the findings are less consistent. Laukka et al. [27] and Pasoglou et al. [26] reported MRI specificity at 100%, whereas CT specificity varied to 75% as reported by Laukka et al. and 100% by Pasoglou et al. However, it is crucial to interpret these results cautiously due to variations in reference standards and study design. Laukka et al. [27] used CT itself as the reference standard, which may have biased the specificity results in favor of MRI. This dependence on CT as a comparator can lead to an overestimation of the specificity of MRI due to the potential for CT to miss smaller or morphologically atypical lymph nodes, which MRI might detect.

Although the evidence does not conclusively show that MRI is superior to CT in specificity, it consistently demonstrates that MRI has comparable diagnostic accuracy to CT. This equivalency is significant because MRI offers the added benefit of avoiding ionizing radiation, making it a valuable alternative, especially for younger patients or in scenarios requiring repeated imaging. However, a key limitation of MRI is that it requires an experienced radiologist to accurately interpret the images, as the interpretation of MRI scans can be challenging and prone to variability depending on the expertise of the radiologist. In addition, MRI is often more costly than CT, which could limit its accessibility, particularly in resource-limited settings. These factors - cost and the need for specialized expertise - should be considered when deciding between the two imaging modalities.

This systematic review has several limitations. First, the inclusion criteria excluded case reports, case series, editorials, commentaries, and reviews, which may have provided additional insights into the topic. Second, the number of articles directly comparing MRI and CT in the detection of retroperitoneal lymphadenopathy was limited, restricting the scope and detail of the analysis. Third, one of the included studies was older [24], potentially affecting the applicability of its findings in the current context. Lastly, meta-analysis faced challenges in combining data from studies that used varying units of analysis, such as patient-level and lesion-level data, potentially leading to variability in the pooled results.

The existing literature highlights the need for further studies that directly compare MRI and CT in detecting retroperitoneal lymphadenopathy. The limited data available suggest that both MRI and CT scans provide comparable diagnostic accuracy, demonstrating high sensitivity in detecting retroperitoneal lymph node metastasis in testicular cancer. This comparability is significant for clinical practice, as it implies that MRI could serve as a viable alternative to CT for imaging during the surveillance of testicular cancer patients, potentially reducing patient exposure to ionizing radiation.

However, MRI has certain limitations, including the need for expert radiologists to interpret the scans accurately and its higher cost, which may restrict its accessibility in some healthcare settings. Our review underscores the importance of conducting more prospective studies using independent reference standards, such as histopathological confirmation, to reliably compare the specificity of MRI and CT in testicular cancer. Future research should also assess cost-effectiveness and the role of radiologist expertise in diagnostic accuracy to establish the full clinical benefits of MRI.

## Conclusions

This systematic review compared the diagnostic accuracy of MRI and CT in detecting retroperitoneal lymph node metastasis in adult males with testicular germ cell tumors. Our findings indicate that both MRI and CT demonstrate high sensitivity, making them valuable tools in clinical practice. While MRI offers the advantage of avoiding radiation, it requires expert interpretation and is associated with higher costs, potentially limiting its accessibility in some settings. The available evidence does not conclusively show that MRI is superior in specificity to CT, highlighting the need for further studies with independent reference standards to confirm these findings.

These insights underscore the importance of our review in guiding clinical decision-making and optimizing imaging strategies for accurately diagnosing retroperitoneal lymph node metastasis in testicular germ cell tumors. By considering the strengths and limitations of each modality, clinicians can make informed choices that balance diagnostic accuracy, cost, and resource availability to best meet patient needs.
